# Supplementary material for: Single Cell Transcriptomic and Chromatin Profiles Suggest Layer Vb Is the Only Layer With Shared Excitatory Cell Types in the Medial and Lateral Entorhinal Cortex
Source: Front Neural Circuits. 2022 Jan 26;15:806154. doi: 10.3389/fncir.2021.806154 (PMC8826650; doi:10.3389/fncir.2021.806154)
Supplement: Supplementary file 14 [file Data_Sheet_1.ZIP › Deep_vs_Sup_Motifs/homerResults/motif3.similar.html]

motif3

## Information for motif3

T
A
G
C
C
G
A
T
A
C
T
G
C
G
T
A
A
C
G
T
C
G
T
A
C
G
T
A
A
T
C
G
  
Reverse Opposite:  

A
T
G
C
C
G
A
T
A
C
G
T
C
G
T
A
A
C
G
T
A
G
T
C
C
G
T
A
A
T
C
G
  

|  |  |
| --- | --- |
| p-value: | 1e-88 |
| log p-value: | -2.046e+02 |
| Information Content per bp: | 1.830 |
| Number of Target Sequences with motif | 3796.0 |
| Percentage of Target Sequences with motif | 40.83% |
| Number of Background Sequences with motif | 10090.3 |
| Percentage of Background Sequences with motif | 30.98% |
| Average Position of motif in Targets | 345.9 +/- 257.2bp |
| Average Position of motif in Background | 345.1 +/- 267.6bp |
| Strand Bias (log2 ratio + to - strand density) | 0.0 |
| Multiplicity (# of sites on avg that occur together) | 1.21 |
| Motif File: | file (matrix) reverse opposite |

### Similar de novo motifs found

|  |  |  |  |  |  |  |  |
| --- | --- | --- | --- | --- | --- | --- | --- |
| Rank | Match Score | Redundant Motif | P-value | log P-value | % of Targets | % of Background | Motif file |
| 1 | 0.805 | T A G C C G T A C G A T C G T A A C G T A G T C A C G T G T C A A C G T G C T A | 1e-81 | -187.668963 | 51.14% | 41.27% | motif file (matrix) |
| 2 | 0.674 | A G C T G T C A A C G T A G T C C G T A G A T C A C G T C G A T | 1e-66 | -152.587331 | 31.24% | 23.41% | motif file (matrix) |
| 3 | 0.676 | C G A T C G A T G C T A C G A T G A T C C T A G C T G A A G C T | 1e-65 | -151.504078 | 34.18% | 26.12% | motif file (matrix) |
| 4 | 0.781 | T G C A A T C G G A C T A G C T T A C G C T G A C G A T T G C A G C T A T C A G | 1e-49 | -113.775978 | 11.56% | 7.25% | motif file (matrix) |
| 5 | 0.692 | C G T A C T A G C T G A A G C T C T G A G T A C A G T C T G C A | 1e-47 | -108.333085 | 21.23% | 15.55% | motif file (matrix) |
| 6 | 0.687 | A C T G C T G A C G A T C G T A C G T A A C T G C G T A A G T C A C T G C G A T A G C T C G T A | 1e-21 | -49.141408 | 22.91% | 18.91% | motif file (matrix) |
| 7 | 0.714 | C A G T C G A T A T G C C A T G C G A T A T C G C G T A C A G T C T G A C G T A | 1e-18 | -42.997889 | 1.90% | 0.90% | motif file (matrix) |
| 8 | 0.678 | T G A C G T C A T A G C T C A G A C G T C A G T G C T A G C A T T G A C G C T A | 1e-10 | -24.386026 | 3.23% | 2.16% | motif file (matrix) |
| 9 | 0.800 | A C T G C G T A A C G T C G T A C G T A A C T G A C G T A G T C | 1e-1 | -3.672366 | 0.81% | 0.64% | motif file (matrix) |
